# Comparative functional morphology indicates niche partitioning among sympatric marine reptiles

**DOI:** 10.1098/rsos.231951

**Published:** 2024-05-15

**Authors:** Davide Foffa, Mark T. Young, Stephen L. Brusatte

**Affiliations:** ^1^ Department of Geosciences, Virginia Tech, Blacksburg, VA, USA; ^2^ School of Geography, Earth and Environmental Sciences, University of Birmingham, Birmingham, UK; ^3^ Department of Natural Sciences, National Museums Scotland, Edinburgh, UK; ^4^ School of GeoSciences, Grant Institute, University of Edinburgh, Edinburgh EH9 3FE, UK

**Keywords:** marine tetrapods, Jurassic, multivariate analyses, morphospace, convergence, faunas

## Abstract

Mesozoic marine ecosystems were dominated by diverse lineages of aquatic tetrapods. For over 50 Ma in the Jurassic until the Early Cretaceous, plesiosaurians, ichthyosaurians and thalattosuchian crocodylomorphs coexisted at the top levels of trophic food webs. We created a functional dataset of continuous craniomandibular and dental characters known from neontological studies to be functionally significant in modern aquatic tetrapods. We analysed this dataset with multivariate ordination and inferential statistics to assess functional similarities and differences in the marine reptile faunas of two well-sampled Jurassic ecosystems deposited in the same seaway: the Oxford Clay Formation (OCF, Callovian–early Oxfordian, Middle–Late Jurassic) and the Kimmeridge Clay Formation (KCF, Kimmeridgian–Tithonian, Late Jurassic) of the UK. Lower jaw-based macroevolutionary trends are similar to those of tooth-based diversity studies. Closely related species cluster together, with minimal overlaps in the morphospace. Marine reptile lineages were characterized by the distinctive combinations of features, but we reveal multiple instances of morphofunctional convergence among different groups. We quantitatively corroborate previous observations that the ecosystems in the OCF and KCF were markedly distinct in faunal composition and structure. Morphofunctional differentiation may have enabled specialization and was an important factor facilitating the coexistence of diverse marine reptile assemblages in deep time.

## 1. Introduction

Jurassic marine ecosystems (*ca* 201–145 Ma) were dominated by three different lineages of reptiles—plesiosaurians, ichthyosaurians and thalattosuchian crocodylomorphs [1–3]—that were secondarily adapted to a marine lifestyle. Quantitative and qualitative studies on the dentition and lower jaws of extinct tetrapods suggest that these animals probably occupied analogous ecological roles nowadays held by cetaceans, large teleosts and sharks [4–11]. Furthermore, stratigraphic and fossil evidence (e.g. bite marks and regurgitates; e.g. [12,13]) suggests that—like their modern counterparts—different lineages coexisted, interacted and perhaps partitioned available resources [6,9,14–17]).

Numerous studies have shown that drastic changes in faunal composition and structure accompanied the Middle–Late Jurassic transition on a global [17–19] and on a local scale (Jurassic Sub-Boreal Seaway, JSBS) [5,20]. Specifically, the latter studies showed a substantial decline in small-body prey specialists and an increase of macrophagous (preying on large food items such as other marine reptiles) taxa across the boundary in the JSBS. These patterns were observed using morphological/functional features of marine reptile dentition because teeth are widely available as fossils, constituting an ideal proxy for feeding ecology [5], and allow for grouping extinct and extant marine tetrapods in feeding guilds (nomenclature after [9]).

It may be expected that that skulls and lower jaws would also undergo modifications consistent with each species’ hypothesized feeding ecology, to accommodate differences in feeding mechanisms and the prey consumed. For example, macrophagous taxa would be expected to have proportionally more robust jaws, than small-bodied prey specialists [21]. Scaling is also an important factor to consider, as larger organisms are capable of feeding on a wider range of prey and can produce more powerful and rapid biting than proportionally identical but smaller species. In other words, it may be possible to derive ecologically informative patterns from analysing the size, shape and mechanical performance of marine reptile lower jaws through time. The macroevolutionary trajectories of plesiosaurs, ichthyosaurs and thalattosuchians have all been individually investigated [18,19,22–29], but rarely are there comparisons across sympatric clades and within the same assemblage. This unexplored gap prevents us from understanding whether different groups converged upon analogous solutions under similar ecological/selection pressures and from investigating broad ecological phenomena (i.e. niche partitioning and competiton).

To explore this gap, we investigate the feeding mechanics and evolution of the marine reptiles of the JSBS using a comparative approach, and then compare the results with those derived from tooth-based studies [5,30]. Because mandibles potentially undergo different selective pressures than teeth, it is crucial to analyse how the shape of lower jaws and crown morphology might covary. Therefore, in this study, we aim to investigate (i) the morphological and functional variation of sympatric marine reptile lower jaws; (ii) whether they are constrained by their evolutionary history (i.e. phylogenetic inertia); and (iii) whether morphological and functional variation in lower jaws could facilitate niche partitioning. The patterns previously found in tooth-based studies provide the basis for testing ecologically informative hypotheses and the foundation to answer both clade-specific and broader ecological questions: are there the functional and morphological combination of characters (aka, morphofunctional complexes) that characterize different phylogenetic groups and feeding guilds? Do distantly related marine reptile clades converge on the same ecological niches in similar ways? And, finally, what morphological and functional changes accompanied each group’s evolution across the Middle–Late Jurassic ecological transitions?

### 1.1. Institutional abbreviations

**BEDFM,** Bedford Museum, Bedford, UK; **CAMSM,** Sedgwick Museum, Cambridge, UK; **DORCM,** Dorset County Museum, Dorchester, UK; **GLAHM,** The Hunterian Museum, Glasgow, UK; **NHMUK,** Natural History Museum, London, UK; **MJML,** Museum of Jurassic Marine Life—the Steve Etches Collection, Kimmeridge, UK; **OUMNH,** Oxford University Museum of Natural History, Oxford, UK; **MJSN,** Jurassica Museum (formerly Musée Jurassien des Sciences Naturelles), Porrentruy, Switzerland; **MNHN,** Muséum National d'Histoire Naturelle, Paris, France; **PETMG,** Peterborough Museum and Art Gallery, Peterborough, UK; **SMNS,** Staatliches Museum für Naturkunde Stuttgart, Germany.

### 1.2. Anatomical abbreviations

**ASD,** gullet size (maximum lateral margin articular distance); **CPD,** maximum dorsoventral depth at the coronoid process/eminence; **eTRD,** dorsoventral depth at the posterior end of the tooth row; **eTRW,** width of the lower jaws at the posterior end of the tooth row; **MA,** mechanical advantage (it can be a,anterior; p, posterior; or lt, at the largest tooth); **ML,** mandibular ramus total length; **MSD,** mandibular symphyseal area maximum dorsoventral depth; **MSL,** mandibular symphysis length; **oMA,** opening mechanical advantage; **maL,** muscle adductor anterior–posterior length; **RPL,** retroarticular process length; **TI,** tooth index; **TRL,** length of the tooth row.

### 1.3. Other abbreviations

**OCF,** Oxford Clay Formation; **JSBS,** Jurassic Sub-Boreal Seaway; **KCF,** Kimmeridge Clay Formation.

## 2. Methods

**Figure 1.**
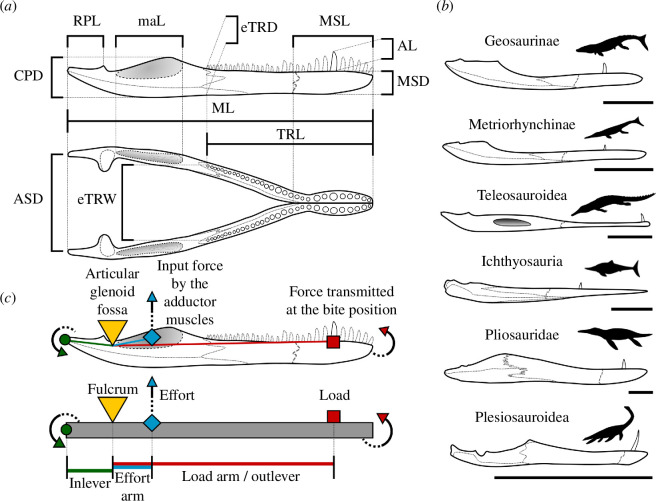
Simplified marine reptile lower jaw and illustration of some of the functional characters used in the principal coordinate analysis (PCoA), morphofunctional and bite performance analyses. (*a*) Measurements used in this study; note that all the measurements have been rescaled to the ML to reduce the effect of size (see electronic supplementary material), which has been accounted for separately. (*b*) Schematic illustration of lever theory applied to a simplified marine reptile lower jaw. (*c*) Comparative plate showing the variations of marine reptile lower jaws in the six groups of the JSBS. From the top to bottom: ‘*Metriorhynchus*’ *cultridens* (= ‘*M*.’ *brachyrhynchus*) (NHMUK PV R 3804); *Thalattosuchus superciliosus* (NHMUK PV R 2030); *Proexochokefalos heberti* (MNHN.F 1890-13); *Ophthalmosaurus icenicus* (based on NHMUK PV R 3893 and GLAHM V1129, redrawn from [31]; *Pliosaurus kevani* (DORCM G.13,675, redrawn from [32]; *Muraenosaurus leedsi* (redrawn from [33]). Scale bars equal 20 cm. See Anatomical abbreviations for the acronyms in the figure.

**Figure 2.**
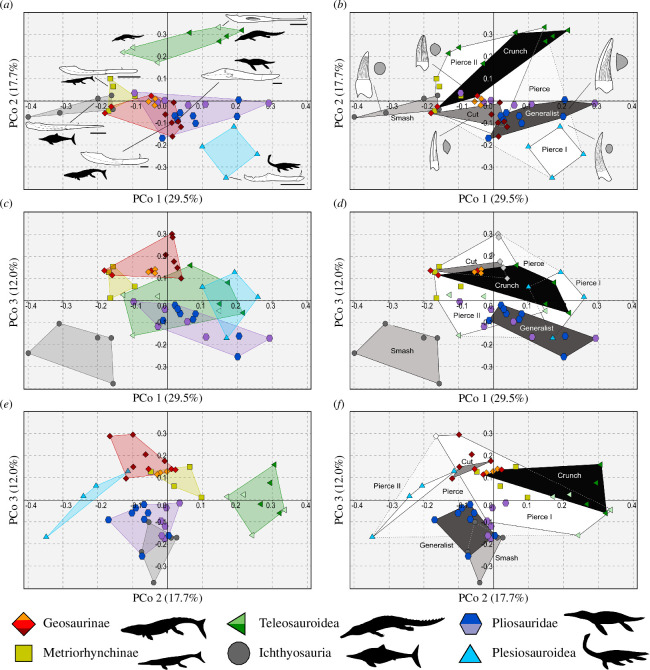
PCoA morphofunctional-morphospace organized by taxonomic groups (left) and tooth-based feeding guilds (right). (*a,b*) PCo1 versus PCo2; (*c,d*) PCo1 versus PCo3; (*e,f*) PCo2 versus PCo3. The line drawings of lower jaws are from *Dakosaurus maximus* (SMNS 82043), *Thalattosuchus superciliosus* (NHMUK PV R 2030), *Proexochokefalos heberti* (MNHN.F 1890–1913), *Ophthalmosaurus icenicus* (based on NHMUK PV R 3893 and GLAHM V1129, redrawn from [31]; *Pliosaurus kevani* (DORCM G.13,675, redrawn from [32]); and *Muraenosaurus leedsi* (redrawn from [33]).

This study specifically focuses on the shape and biomechanics of marine reptile lower jaws (figure 1). Our dataset consists of 47 of the most complete and best-preserved lower jaws from the Callovian–Tithonian interval of the JSBS (approx. 166–145 Ma). A handful of taxa from the UK have incomplete lower jaws, so for these, we used more complete congeneric specimens from contemporaneous formations of Europe (i.e. France, Germany and Switzerland). Specifically, we extracted data from the lower jaws of *Torvoneustes jurensis* (MJSN BSY008-465; [34]); *Proexochokefalos heberti* (MNHN.F 1890-13) *Machimosaurus buffetauti* (SMNS 91415), *Suchodus durobrivensis* (unnumbered specimen; see [35]) and *Dakosaurus maximus* (SMNS 82043). The measurements of the majority of specimens were personally hand measured by D.F. with digital callipers, while the remaining were obtained through literature and corroborated with digital measurements using ImageJ [36]. Mandibular measurements were preferred over cranial ones because the former have a clearer functional significance and can be better correlated to diet and ecological niches in extant and extinct taxa [10,37–39]. In order to avoid character overlaps with the dental datasets in Foffa *et al*. [5] only a single tooth-related character (TI) was included in this study. We decided to adopt this strategy so that no character is shared with the tooth-based analyses of Foffa *et al*. [5]. Because these two analyses are independent, it allows us to separately assess the importance of mandibular features in determining guild partitioning. Eleven measurements were selected and standardized (*z*-corrected) before being used in a principal coordinate analysis (PCoA; Euclidean similarity index) that we used to investigate the overall functional morphology of marine reptile lower jaws. Data points were organized first by phylogenetic groups and, separately, by tooth-based feeding guilds (figure 2), which were assigned *a priori* based on quantitative testing of these guilds in previous studies [5]. Note that the majority of taxa and specimens used in this study were also included in Foffa *et al*. [5]. We also ran a series of in-depth comparative analyses of specific features to better compare the morphology and the function (bite performances) of marine reptile lower jaws across-clade (see §2.5).

### 2.1. Taxonomic sampling and phylogenetic groups

This study differs from previous quantitative works in the taxonomic spread (i.e. across-clade comparisons) and finer time resolution subdivision. Specifically, marine reptiles were assigned into family/subfamily groups allowing tests of ecological differences among and within groups. It is wellknown that a large variety of morphologies is present within the same Order, most evidently exemplified by group-focused studies, particularly on Plesiosauria and Thalattosuchia [5,26–28,39]. Accordingly, adopting lower-tier taxonomic groupings, and a set of characters that is applicable to all of them, increases the resolution of the functional analyses and can test evolutionary patterns both within and across groups. A total of 47 specimens, representing 43 described taxa and 4 that are currently undescribed or indeterminate, were grouped as follows: 14 pliosaurids, 4 plesiosauroids, 16 metriorhynchids (4 metriorhynchines and 12 geosaurines), 8 teleosauroids and 5 ichthyosaurians (electronic supplementary material, appendix S1). No juvenile specimens were included unless they are the unique occurrence of a taxon otherwise unrepresented in this dataset.

Sample size limited our choice to subdivide groups in lineages. Thus, most of the analyses compared the following groups: Plesiosauroidea, Pliosauridae, Metriorhynchinae, Geosaurinae, Ophthalmosauridae and Teleosauroidea, but a finer subdivision was sometimes possible. However, we also discussed patterns in the following evolutionary important sub-lineages: three pliosaurids lineages, namely, small-bodied longirostrine taxa (*Peloneustes, Marmornectes* and *Eardasaurus*); mid–large-bodied Thalassophonea (*Liopleurodon, Simolestes* and ‘*Pliosaurus*’ *andrewsi*); and species of the genus *Pliosaurus*. Within Metriorhynchidae, we considered three geosaurine lineages: basal geosaurines (only including ‘*Metriorhynchus*’ *brachyrhynchus*), T*-*clade (*Tyrannoneustes, Torvoneustes*, Mr. Passmore’s specimen*,* cf. ‘*Metriorhynchus*’ *hastifer* and their closest relatives); GPD-clade (*Geosaurus, Plesiosuchus, Dakosaurus* and their closest relatives); and metriorhynchines. Two lineages of teleosauroids were discussed: non-machimosaurin teleosauroids (*Charitomenosuchus leedsi* and *Mycterosuchus nasutus*), including the ecologically intermediate *Proexochokefalos heberti* and *Neosteneosaurus edwardsi*; and Machimosaurini. It was not possible to further subdivide Ichthyosauria and Plesiosauroidea.

### 2.2. Guild system

Alongside phylogenetic-based groups, we ran a version of each analysis subdividing our sample into feeding guilds based on tooth morphology ([5], but also see [4,9]). We consider a version of such system that includes six groups: Cut Guild; Generalist Guild; Pierce Guilds—subdivided into Pierce I and Pierce II; Smash Guild; Crunch Guild. Tooth shape of extant and extinct marine tetrapods is a reliable indicator of diet [9], giving further ecological meaning to our analyses.

### 2.3. Time binning

**Figure 3.**
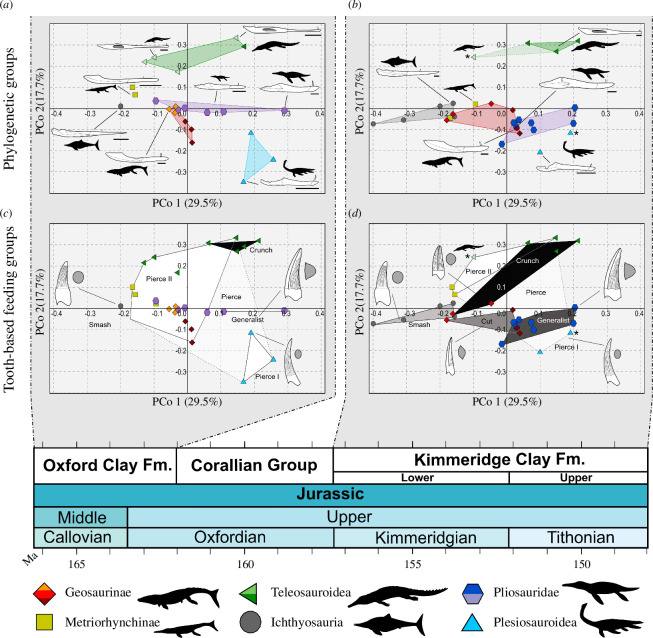
PCoA functional-morphospace plots comparing the morphospace occupancy of marine reptile clades (*a,b*) and tooth-based feeding guilds (*c,d*) between the OCF (***a,c***) and KCF (***b*,*d***). The line drawings of lower jaws are from top to bottom: (***a***) *Proexochokefalos heberti* (MNHN.F 1890–1913); *Mycterosuchus nasutus* (reconstruction based on CAMSM J.1420 and NHMUK PV R 2617); *Thalattosuchus superciliosus* (NHMUK PV R 2030); *Peloneustes philarchus* (based on CAMSM J.46913 and NHMUK PV R 3803); *Simolestes vorax* (based on NHMUK PV R 3319 and NHMUK PV R 3170); *Ophthalmosaurus icenicus* (based on NHMUK PV R 3893 and GLAHM V1129, redrawn from [31]; ‘*Metriorhynchus*’ *cultridens* (= ‘*M*.’ *brachyrhynchus*) (NHMUK PV R 3804); *Muraenosaurus leedsi* (based on a reconstruction of NHMUK PV R 2422 and NHMUK PV R 2421, redrawn from [33]; and (***b***) *Machimosaurus* sp. (based on SMNS 91415); *Brachypterygius extremus* (based on CAMSM J.68516); *Pliosaurus kevani* (DORCM G.13,675, redrawn from [32]; *Dakosaurus maximus* (SMNS 82043); *Kimmerosaurus langhami* (based on [40] reconstruction of NHMUK PV R 8431).

The lack of sufficiently well-preserved specimens, and inadequate locality and stratigraphic information, allows only for a coarse subdivision into two time-averaged assemblages (figure 3). So, while this subdivision does not punctually capture a single snapshot of an ecosystem at one point in time, it is sufficient to investigate broad differences between the Oxford Clay Formation (OCF) and Kimmeridge Clay Formation (KCF), the richest assemblages before and after the Middle–Late Jurassic transition in the JSBS. Of the 47 specimens included in the dataset, 25 come from the OCF and 22 from the KCF.

### 2.4. Principal coordinate analysis

The functional morphology of the 47 marine reptiles' lower jaws was investigated using a dataset of 11 continuous characters of documented morphofunctional significance [6,10,26,37,39] (electronic supplementary material, appendices S1 and S2; table 1). Seven lower jaw measurements were standardized to mandibular length (ML) (figure 1) (electronic supplementary material). Standardized measurements and mechanical advantage metrics were then normalized (brought to the same mean and standard deviation—*Z*-correction) to account for size variation [5,39] (electronic supplementary material). The complete dataset was analysed using PCoA in PAST v. 4.12 [41] and R v. 4.3.1 [42].

**Table 1.** Description of the characters used in the PCoA.

character	abbreviation	description
Ch.1	ASDm/ML	relative gullet size
Ch.2	MSL/ML	relative length of the mandibular symphysis
Ch.3	MSD/ML	relative depth of the mandibular symphysis
Ch.4	TRL/ML	relative length of the tooth row
Ch.5	CPD/ML	maximum depth of the mandible (at the coronoid process) relative to total length
Ch.6	RPL/ML	relative length of retroarticular process
Ch.7	TI	tooth index
Ch.8	maL/ML	relative length of the muscle adductor insertion
Ch.9	aMa	anterior mechanical advantage
Ch.10	pMA	posterior mechanical advantage
Ch.11	MA	opening mechanical advantage

The separation of groups in the PCoA functional morphospace was investigated using one-way permutational multivariate analyses of variance (PERMANOVA) on all 11 principal coordinate (PCo) axes. We adopted the null hypothesis that groups are not significantly separated (no difference in the location of group centroids) in the PCoA. However, it was not possible to run one-way PERMANOVA tests for every subgroup comparison because of the limited sample sizes of some lineages (e.g. Plesiosauroidea, Metriorhynchinae, Ichthyosauria and the two groups within Teleosauroidea) (electronic supplementary material, appendix S3), but such comparisons were possible at higher taxonomic ranks (including some between time bins) and guilds. To account for ‘false rate discoveries’ errors (introduced when conducting multiple comparisons) the *p* values were adjusted using the false-discovery rate [43] using R [42]. Any deviation from the null hypothesis (Bonferroni-adjusted *p* values < 0.05) indicates that the groups occupy significantly different areas of the PCoA morphospace using R [42].

**Figure 4.**
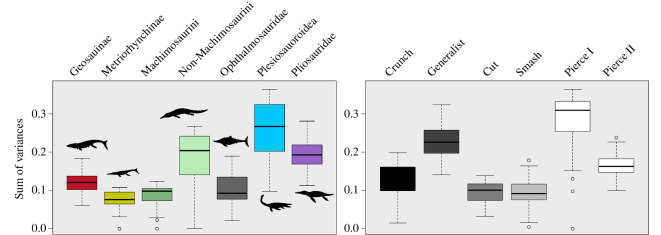
Box plot sum of variances comparing the lower jaw morphofunctional disparity of marine reptiles subdivided into taxonomic groups and tooth-based feeding guilds.

The PCo morphospace was generated using PAST v. 4.12. We performed PERMANOVA pairwise comparison to assess for significant separations of groups in the morphospace in R [42]. We also performed permutation tests to assess statistically significant differences in disparity (morphofunctional variety measured in sum of variances) between groups, and within groups across time based on PCoA axis values. The codes we used were developed by Wang [44] and modified to incorporate functions of the ‘dispRity’ package [45] in R (code available in [5]; electronic supplementary material). We also additionally performed Bonferroni-corrected Wilcox tests on the disparity data (sum of variances) using another code developed by D.F. (figure 4) (electronic supplementary material). Finally, we tested the predictive power of lower jaw morphofunctional characters in classifying our taxa based on phylogenetic groups and different tooth-based guilds, using linear discriminant analyses (LDA) in PAST v. 4.12.

### 2.5. Comparisons of morphological and functional features

**Figure 5.**
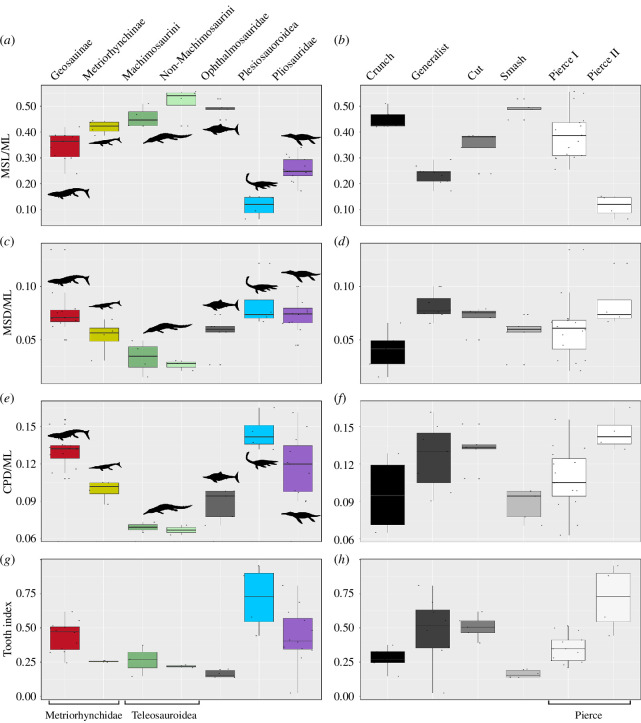
Box plot of morphological metrics comparing the lower jaws of marine reptiles subdivided into taxonomic groups and tooth-based feeding guilds.

**Figure 6.**
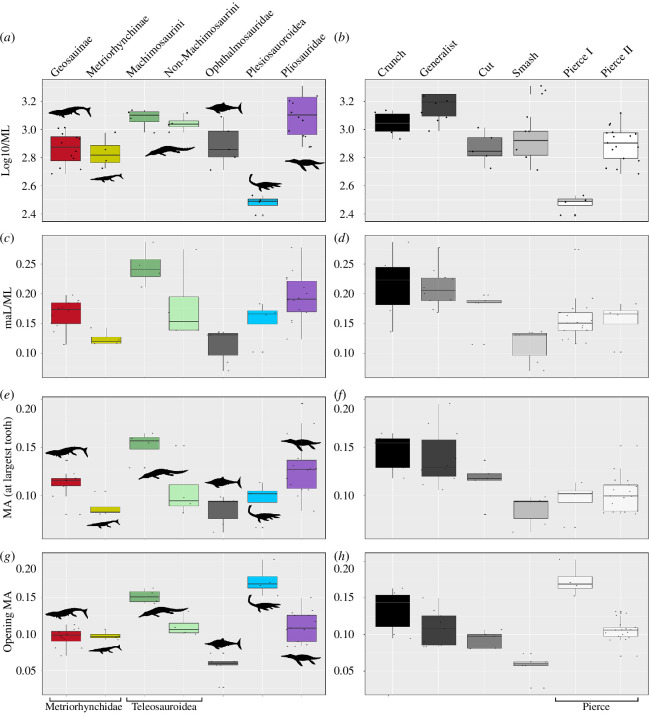
Box plot of functional metrics comparing the lower jaws of marine reptiles subdivided into taxonomic groups and tooth-based feeding guilds.

**Figure 7.**
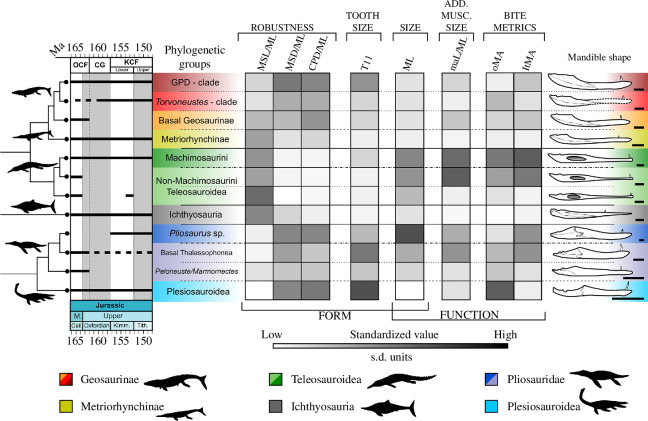
Marine reptile lower jaw comparative table based on functional-morphological characters. The line drawings of lower jaws are from the top to bottom: *Dakosaurus maximus* (SMNS 82043), *Torvoneustes coryphaeus* (MJML K1863); ‘*Metriorhynchus*’ *cultridens* (= ‘*M*.’ *brachyrhynchus*) (NHMUK PV R 3804); *Thalattosuchus superciliosus* (NHMUK PV R 2030); *Machimosaurus* sp. (based on SMNS 91415); *Proexochokefalos heberti* (MNHN.F 1890–1913); *Mycterosuchus nasutus* (reconstruction based on CAMSM J.1420 and NHMUK PV R 2617); *Ophthalmosaurus icenicus* (based on NHMUK PV R 3893 and GLAHM V1129, redrawn from [31]); *Pliosaurus kevani* (DORCM G.13,675, redrawn from [32]); *Simolestes vorax* (based on NHMUK PV R 3319, NHMUK PV R 3170); *Peloneustes philarchus* (based on CAMSM J.46913, NHMUK PV R 3803); *Muraenosaurus leedsi* (based on a reconstruction of NHMUK PV R 2422, NHMUK PV R 2421, redrawn from [33]). Scale bars equal 10 cm. See Anatomical abbreviations for the acronyms in the figure.

**Figure 8.**
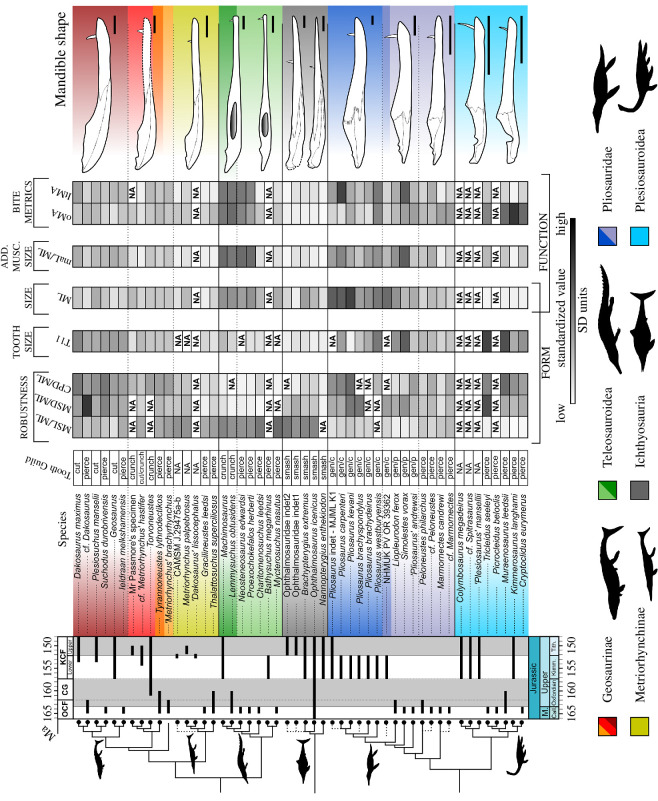
Marine reptile lower jaw species-level comparative table based on functional–morphological characters. The line drawings of lower jaws are from top to bottom: *Dakosaurus maximus* (SMNS 82043); *Torvoneustes coryphaeus* (MJML K1863); ‘*Metriorhynchus*’ *cultridens* (= ‘*M*.’ *brachyrhynchus*) (NHMUK PV R 3804); *Thalattosuchus superciliosus* (NHMUK PV R 2030); *Machimosaurus* sp. (based on SMNS 91415); *Mycterosuchus nasutus* (reconstruction based on CAMSM J.1420 and NHMUK PV R 2617); *Brachypterygius extremus* (based on CAMSM J.68516); *Ophthalmosaurus icenicus* (based on NHMUK PV R 3893 and GLAHM V1129, redrawn from [31]); *Pliosaurus kevani* (DORCM G.13,675, redrawn from [32]); *Simolestes vorax* (based on NHMUK PV R 3319, NHMUK PV R 3170); *Peloneustes philarchus* (based on CAMSM J.46913, NHMUK PV R 3803); *Muraenosaurus leedsi* (based on a reconstruction of NHMUK PV R 2422, NHMUK PV R 2421, redrawn from [33]); *Kimmerosaurus langhami* (based on [40] reconstruction of NHMUK PV R 8431). Scale bars equal 10 cm. See Anatomical abbreviations for the acronyms in the figure.

In addition to the undifferentiated PCoA morphofunctional analysis, we isolated some of the most relevant morphological and functional metrics for direct comparisons (figures 5–8). We used this approach for two reasons. Firstly, to detect instances of functional convergence: the same functional outcome can be obtained through distinct morphological routes that could be difficult to discern in the PCoA. Secondly, direct comparisons of individual features allow us to inspect more clearly whether there are repeated patterns within phylogenetic and ecological groups (e.g. shortening of mandibular symphysis in all macrophagous taxa, reduction of tooth size in piscivore specialists, etc.). To achieve this, we separated morphological features from more strictly functional ones (figures 5 and 6). The metrics we selected to describe morphology are proxies to size (ML), robustness (MSL/ML; MSD/ML; CPD/ML) and relative tooth sizes (TI1) (figures 1 and 5–8; see electronic supplementary material).

— *Size*. The standardization and deliberate use of non-dimensional characters in the PCoA largely remove the impact of body size from the PCoA distribution. However, sheer dimension is a well-known factor that contributes to the mechanical performance of any (biological or artificial) structure. For example, given a large mandible and its downsized exact copy, the former will resist more mechanical stress than the latter—increasing bone area and mass, axiomatically reduces stress force per unit area (and thus increasing mechanical resistances). Thus, in feeding mechanics, large size may be considered advantageous for macrophagous taxa even in the absence of specific strengthening morphological features. Comparatively large jaws and teeth confer other benefits, as they increase the likelihood of successful predation (larger surface to catch prey), and the efficiency of tooth-cutting edges [46,47]. On the downside, ML has also been suggested to be a proxy for maximum gape, which is thought to decrease bite force-related performance [10,37,39,48–51]. In marine reptile studies, this pattern of increased body size is suggested to be an important factor, for example, in macrophagous pliosaurids [21,26,38,52–54]. Although it is not strictly involved in the mechanics of jaws, size is linked to many factors considered in it (e.g. gape, input force, mechanical resistances, maximum prey size) and cannot be ignored*.*
— *Robustness*. Here, robustness characters specifically refer to the proportions of the mandibular symphysis (MSL/ML and MSD/ML) and maximum depth of the lower jaw, normally at the coronoid process (CPD/ML). These features describe in first approximation the cross-sectional area of the lower jaw, a key factor to determine the mechanical resistance to bending and torsion in the absence of more reliable measurements of I (second moment of area) and J (polar moment of inertia) [39,55,56]. In extant crocodylians and toothed cetaceans, gracile lower jaws with long shallow mandibular symphyses are associated with feeding on small-bodied prey items caught with agile lateral movements [52,57–59]. In other words, long and shallow jaws perform worse under regimes of high-stress feeding (e.g. macrophagy, durophagy), than deeper jaws of the same size. These patterns have been observed and tested using various techniques in many extinct and extant aquatic tetrapods including metriorhynchids [29,39,59].
— *Tooth index*. Numerous studies have demonstrated the importance of the relative size of teeth compared with the lower jaws [6,9]. Of the numerous tooth indices, here only TI1—the relative size of the largest tooth against the jaw length—is used, because it was available for a larger number of taxa.

Similarly, we selected three other metrics to compare functional performances across groups and tooth-based feeding guilds (figures 5–8). These are proxies for input force (maL/ML), force transmission at the largest tooth (ltMA) and bite speed (oMA):

— *Input force*. The force exerted by muscle groups is proportional to their cross-sectional area (CSA [60]. The relative length (compared with the total mandible length—maL/ML) of adductor muscle insertion sites on the lower jaw was used instead of the CSA, which is unmeasurable in deformed or incomplete fossils. It is worth noting that pennation is a significant factor for muscle contractile forces, which unfortunately cannot be taken into account in this analysis. Ignoring muscle pennation has been put forward as a reason why biomechanical models often underestimate bite forces [61]. Nevertheless, in these comparative analyses, maL/ML (which unlike pennation can be measured in extinct taxa) is used as a proxy for adductor muscle force.
— *Force transmission at biting point*. In a lever system, the mechanical advantage MA quantifies the amount of input force transferred to the output. In a jaw system, it measures the quantity of adductor musculature force transferred to the bite position. For this analysis, the bite position was chosen and calculated for each taxon at the largest tooth position [62] (see electronic supplementary material).
— *Opening speed*. Opening mechanical advantage is the ratio between an output lever arm and an input lever arm which extends beyond the fulcrum (figure 1). This index quantifies the ‘efficiency’ of the jaw depressor muscles, providing a proxy for jaw-opening speed. It also gives insight on feeding patterns and prey selection [63] (see electronic supplementary material).

Absolute and standardized values can also be found in the electronic supplementary material, appendices S1 and S2. Pairwise *t*-tests and Wilcox (Bonferroni corrected) were run to determine statistical differences in these metrics among phylogenetic groups and tooth-based feeding guilds (figures 5 and 6). To facilitate visualization, all these metrics were standardized across all species, and different groups were plotted using the heatmap function of the phytools package v. 2.0-3 [64] (figures 7 and 8) alongside informal phylogenetic trees and respective tooth guild.

## 3. Results

### 3.1. Principal coordinate analysis

#### 3.1.1. Interpretation of the principal coordinate analysis space

The first three PCo axes that describe approximately 60% of the overall variance of the dataset (PCo1 = 29.5%, PCo2 = 17.7%, PCo3 = 12.0%) were used to represent the functional space (figures 2 and 3). All 11 PCo axes were retained for statistical testing (electronic supplementary material, appendices S2–S4). The main characters driving the distribution of taxa along PCo1 are linked to jaw mechanics (i.e. aMA, pMA, oMA and maL/ML), and the length of the retroarticular process (RPL/ML). The distribution of taxa along PCo2 is primarily determined by characters that describe the robustness of the lower jaws (i.e. CPD/ML; MSD/ML); the length of the tooth row is the primary factor along PCo3 (figures 1–3).

Overall, the jaws that characterize species that are interpreted as fast-prey specialists such as metriorhynchines and ichthyosaurians occupy the negative region of PCo1, while the ‘slower’ and more bite-efficient mandibles of pliosaurids, machimosaurins and plesiosauroids cluster on the positive side of the same axis (figures 2 and 3). Similarly, the comparatively slender (longer and shallower) mandible of teleosauroids occupy the positive quadrants of PCo2, while the relatively shorter and deeper lower jaws of plesiosauroids, geosaurines and pliosaurids are all found along negative PCo2 (figures 2 and 3). Ichthyosaurs occupy the negative extremes of PCo3 as opposed to geosaurines, which occupy the positive end of it. This interpretation of the morphospace informed the main characters that we selected for direct comparisons (figures 2 and 3).

#### 3.1.2. Taxonomic groups

The majority of phylogenetic groups in the PCoA morphospace occupy a distinct region relative to another (both overall and in each of the two-time bins) as shown by the results of the one-way PERMANOVA tests (figures 2 and 3; table 2; electronic supplementary material, appendix S4). The result of the one-way PERMANOVA tests shows that all marine reptile clades occupy significantly distinct regions of the PCoA space (table 2; electronic supplementary material, appendices S4 and S7). Plesiosaurians and teleosauroids have the largest disparity among groups, significantly higher (*p* values < 0.001) than that of metriorhynchids and ophthalmosaurids (figure 4; electronic supplementary material, appendix S7). Pliosaurid disparity in the OCF is significantly different from that in the KCF (*p*‐value = 0.002), in confirmation of historically observed patterns [18,26,32,65,66]. It was not possible to detect instances of migration through the PCo space between the OCF and KCF because of the insufficient sample size of the groups once split into time bins. The distribution of species within their phylogenetic group mimics the pattern of the overall PCo just described above: lineages characterized by fast jaw-opening clusters along smaller PCo1 values, while those characterized by more bite-efficient ones are distributed along smaller PCo2 values (figures 2 and 3).

**Table 2.** Result of PERMANOVA tests among marine reptile clades. Statistically significant differences in Bonferroni-corrected *p* values are highlighted in italics.

	Pliosauridae	Plesiosauoroidea	Metriorhynchidae	Teleosauroidea	Ophthalmosauridae
Pliosauridae		*0.003*	*0.001*	*0.001*	*0.001*
Plesiosauoroidea	*0.003*		*0.006*	*0.015*	0.068
Metriorhynchidae	*0.001*	*0.006*		*0.001*	*0.001*
***Teleosauroidea***	*0.001*	*0.015*	*0.001*		*0.024*
Ophthalmosauridae	*0.001*	0.068	*0.001*	*0.024*	

In our analysis, we uncovered similar patterns to those found in our previous tooth-based analyses [5] across the Middle to Late Jurassic. In brief, the taxonomic groups occupy distinct areas of the morphospace with minimal overlap. The transition from the OCF to KCF shows an evident increase in disparity for ichthyosaurs and geosaurines, and a shift in morphospace occupation in pliosaurids (moving towards an area of the PCo space that represents more efficient biting and more robust lower jaws). Similarly, there is an increase in machimosaurin teleosauroid diversity, although there is evidence that longirostrine teleosauroid morphologies were also maintained (although too incomplete to be included in this dataset) [5,27].

The jackknifed LDA correctly classify approximately 89–98% of our data points to their correct phylogenetic group, depending on groupings, with only a minor discrepancy within metriorhynchids.

#### 3.1.3. Feeding guilds

Taxa in the Smash Guild (i.e. ichthyosaurians) occupy an area of negative PCo1–PCo2–PCo3 morphospace (e.g. gracile jaws with low mechanical advantage and moderately sized adductor musculature), indicating a distinct functional morphology from the other groups (figures 2 and 3). Taxa of the Smash Guild cluster close to some of the Pierce II Guild (metriorhynchines and small-bodied longirostrine pliosaurids), but relatively distant from longirostrine teleosauroids of the same guild. Small-bodied pliosaurids of the OCF of the Pierce II Guild (e.g. *Peloneustes*, *Marmornectes* and *Eardasaurus*) cluster separately from the Generalist, macrophagous pliosaurid species (*Simolestes*, *Liopleurodon* and ‘*Pliosaurus*’ *andrewsi*). This separation occurs along PCo2, corroborates the separation based on feeding guilds of these taxa—a hypothesis that is also supported by biomechanical results (see §4). Similarly, longirostrine teleosauroids of the Pierce Guild cluster away from their durophagous relatives (i.e. Machimosaurini), occupying an isolated positive PCo2 area that is characterized by higher mechanical advantage values and increased adductor muscle insertion site size (figures 2 and 4). The Pierce I and Generalist guilds have the highest disparity (sum of variances) among all feeding guilds (figure 4).

Comparing the OCF with the KCF (figure 3) shows a drastic increase of macrophagous (Cut, Crunch and Generalist) and Smash guilds disparity through time, and a strong reduction in Pierce guilds, as previously shown by Foffa *et al*. [5]. All these changes are accompanied by a shift in taxonomic composition in some guilds such as the disappearance of geosaurines from the Pierce guilds and their entering in the Crunch Guild [5].

The jackknifed LDA correctly classify approximately 70–80% of our data points to their *a priori* tooth-based feeding guild. The predictive power of the PCo is lowest when the Pierce guilds are kept unified and highest when Pierce I and Pierce II are kept separate.

### 3.2. Comparisons of morphological and functional features

Comparisons of morphological and functional features broadly confirm the PCoA patterns, but also add specific details. For example, among the morphological characters we selected, the length of the mandibular symphysis (MSL/ML) and mandibular maximum relative depth (CPD/ML) are the features that tend to separate groups, particularly among unrelated phylogenetic groups. Characters related to the depth of the mandible (CPD/ML and MSD/ML) have a much more restricted spread and, interestingly, statistically indistinguishable distributions are shared among species belonging to different tooth-based feeding guilds (figures 5–8; electronic supplementary material, appendix S6).

ML (our proxy for size) is effective in distinguishing both phylogenetic groups and tooth-based feeding guilds, except for taxa commonly regarded as macrophagous, and the difference between certain guilds (e.g. Crunch versus Smash; and Generalist versus Cut versus Pierce II). Unsurprisingly, plesiosauroid size is statistically smaller than that of other groups. Similarly, the size of metriorhynchids (both metriorhynchines and geosaurines) and pliosaurids are significantly different. In confirmation of previous qualitative studies, the size distributions of pliosaurids sizes are significantly distinct in the OCF and KCF, a pattern that is not mimicked by any other group in our dataset.

Among the functional characters, some guilds (Cut, Generalist and Crunch) have lower jaws that are better at transmitting force at the bite position (higher values of aMA, pMA and ltMA) than members of other feeding guilds, but this does not appear to be unique to specific clades, if we exclude extremes (e.g. machimosaurins versus ophthalmosaurids). Perhaps surprisingly, the transmission of muscle force at the biting point of large-bodied pliosaurids is not as high as that of macrophagous teleosauroids (machimosaurins).

Overall, our analyses reveal multiple instances of morphological and functional convergence (expressed in the repeated patterns of changes in values of one or more characters compared with those of close relatives) among distantly related clades in the same feeding guild (figures 7 and 8; table 3; electronic supplementary material, appendix S6). For instance, macrophagous (i.e. Generalist, Cut and Crush guilds) taxa, such as large-bodied thalassophoneans, derived geosaurine metriorhynchids and machimosaurins are characterized by more robust jaws, comparatively larger teeth and adductor muscle sites than their close relatives in other guilds. Similarly, the thalattosuchians and small-bodied pliosaurids belonging to the Pierce I Guild, all have relatively longer, gracile jaws and smaller muscle insertion site areas than their close relatives in other guilds. It is, however, taxa in the Smash Guild (i.e. ichthyosaurians) that have the most gracile lower jaws with the lowest TI among marine reptiles of the JSBS. This corroborates the results of qualitative as well as phylogenetically focused studies. For example, the transition between longirostrine and durophagous teleosauroids is accompanied by the relative shortening of the mandibular symphysis, and increased size of muscle insertion sites and tooth crowns [27]. Similarly, members of the Smash and Pierce guilds are generally the smaller taxa in their taxonomic groups, and bite speed (low oMA) is the most important factor in their bite performance. Accordingly, the lower jaws of *Peloneustes*/*Marmornectes*, metriorhynchines and non-Machimosaurini teleosauroids were faster to open underwater than those of larger thalassophoneans, geosaurines and machimosaurins (figures 5–8; table 3; electronic supplementary material, appendix S5). This speed depression advantage, however, corresponds to a lower muscle contractile force magnitudes being transmitted to the bite points (i.e. a lower bite force), because of the adductor muscle insertion sites being smaller in volume in these taxa—a feature that reduces the bite force (as the in-lever gets shorter).

**Table 3.** Marine reptile lower jaw morphofunctional comparative table.

					mechanical advantage	
**groups**	**tooth guild**	**ML range (mm**)	**robustness**	**MSL (% of total ML**)	**efficiency of force transmission (at largest tooth**)	**jaw-opening speed**
Geosaurinae—Basal	Pierce	~500–800	medium	30–40%	medium	medium to fast
Geosaurinae*—*T*-*clade	Crunch/Cut (cf. ‘*M.’ hastifer*)	~500–1100	medium to deep	20–40%	medium to low	slow
Geosaurinae*—*GPD*-*clade	Cut	~500–1100	deep	20–40%	medium	fast
Metriorhynchinae	Pierce (II)	~500–950	shallow	40–45%	low	fast
Longirostrine teleosauroids	Pierce (II)	~950–1370	very shallow	42–55%	low	fast
Machimosaurini	Crunch	~950–1325	shallow	42–50%	high	slow
Ichthyosauria	Smash	~630–1230	shallow	45–55%	low	very fast
Pliosauridae*—Peloneustes/ Marmornectes*	Pierce (II)	~700–900	shallow to medium	17–35%	low to medium	medium
Pliosauridae—basal Thalassophonea	Generalist Pierce	~800–1500	medium	20–30%	high	medium
*Pliosaurus* sp.	Generalist Cut	~1200–2050	medium to deep	20–30%	high	medium to fast
Plesiosauroidea	Pierce (I)	~250–350	medium to deep	5–15%	very low	slow

Plesiosauroids are a unique case in that they have the least effective bite transmission mechanisms, their jaws are the shortest and slowest. Specifically, in the Pierce II Guild, plesiosauroids fall in an area of the morphospace defined by the shortest mandibular symphysis length, highest TI and highest MA values as well as the smallest jaw length in our dataset (figures 2, 5 and 6). This suggests that plesiosauroids adopted a unique foraging strategy [15,33,67], different from that of other taxa in the Pierce Guild (i.e. the ‘trap guild’ proposed by Chatterjee and Small [68]. This separation supports the original subdivision in Pierce I and Pierce II by Massare [9], a split that could not be confirmed by the dentition-only study by Foffa *et al*. [5].

## 4. Discussion

Results of the PCoA show that each marine reptile group has a unique suite of lower jaw morphofunctional characters (table 2). The comparative morphological and bite performance analyses show a repetition of analogous trends for taxa sharing similar feeding ecologies (figures 5–8; table 3; electronic supplementary material, appendix S5). Taxa assigned to the same feeding guild based on tooth morphology are broadly characterized by similar sets of morphofunctional features, as demonstrated by our statistical analyses (figures 5–8; table 3; electronic supplementary material, appendices S4 and S5), relative to the variation within their phylogenetic group. Consequently, even though all groups are significantly separated in the PCoA (table 2), taxa of similar feeding guild tend to cluster in the same region of the morphospace compared with their close relatives in other guilds (figure 2 and 3; electronic supplementary material, appendix S4). A close examination of the comparative morphological and functional analyses shows that analogous morphofunctional complexes characterize taxa with the same inferred ecology (figures 5–8; table 3; electronic supplementary material, appendices S5 and S6).

There are three exceptions to this general pattern:

— Plesiosauroids cluster away from the other groups in the Pierce Guild. Plesiosauroid morphological and functional features (smallest jaw size in the sample, highest MA and TI) indicate that they had a distinct feeding ecology compared with the other members of the Pierce Guild. We argue that this result shows a clear limitation of basing ecological inference solely on tooth morphology.
— Machimosaurin and geosaurine thalattosuchians of the Crunch Guild occupy distinct areas of the morphospace. The separation between these two clades in the PCoA may indicate that these lineages converged to the same guild through different functional paths.
— *Proexochokefalos heberti*, the only large-bodied teleosauroid in the Pierce II Guild, clusters together with Machimosaurini of the Crunch Guild (see §4). This result was extensively discussed by Foffa *et al*. [5] and in detail by Johnson *et al*. [27], who showed that tooth specialization in Machimsaurini lagged behind mandibular adaptations linked to consuming large-bodied prey items. Similarly, we notice that geosaurines of the GDP-clade evolved their morphofunctional complex in advance of the dental adaptations that place them in the Cut guild. Overall, our results may indicate that dental and lower jaw evolution is decoupled, with specialist tooth morphology often lagging behind mandibular functional specialization (figures 5 and 6; table 3; electronic supplementary material, appendix S4).

### 4.1. Marine reptile functional diversity through time

Morphofunctional differentiation determines the separation among groups in the PCoA morphospace (figure 2). Here, we discuss the functional variations in each group before discussing their changes through time.

#### 4.1.1. Plesiosauria

##### 4.1.1.1. Plesiosauroidea

Plesiosauroid lower jaws are considerably different from all other marine reptiles, including pliosaurids—their closest relatives within Plesiosauria. Plesiosauroid jaws are characteristically small, all in the range of 20–30 cm, approximately half the size of the smaller taxa of any other group. Plesiosauroids (except for *Kimmerosaurus*) have the highest TI (with teeth with the highest crown base ratio, CBR) in our sample of marine reptiles (figures 5, 7 and 8). Although this dental feature has been used to support their belonging to the Pierce Guild, our analyses indicate that plesiosauroids are morphofunctionally distinct from all other taxa in that guild. Plesiosauroids have a combination of a short symphysis, long tooth row and comparatively small adductor muscle size, and they have the highest jaw-opening MA values, combined with the lowest mechanical advantage (i.e. the least effective force transmission to bite position) (table 1; figures 6–8). The high TI and the jaw mechanics fit well with Massare’s [9] proposal that plesiosauroid teeth were well suited to pierce soft prey items. However, other than the shape and proportions of their teeth, plesiosauroids lack any other morphofunctional features that are common in the other members of the Pierce Guild (i.e. fast-opening jaws, long mandibular symphysis, gracile jaws, small adductor musculature relative to mandible length). The distinction between plesiosauroids and other members of the Pierce Guild was observed based on qualitative data by Chatterjee and Small [68], who proposed the addition to the new category of ‘Trap Guild’.

The documented presence of *Muraenosaurus*-like teeth in the KCF [40] may indicate that the feeding mechanics of plesiosauroids were maintained without dramatic modifications across the Middle–Late Jurassic boundary [5,20,40]. *Kimmerosaurus langhami* might be an exception to this pattern, as this taxon could diverge from the traditional plesiosaur feeding style based on the unusual shape and a high number of strongly hooked teeth [40,69]. Similarities with *Morturneria seymourensis—*a Late Cretaceous elsamosaur—indicate that both taxa could be adapted for filter feeding [68]. In support of this hypothesis, this taxon has comparatively smaller muscle insertion areas compared with its closest relatives (figure 8; electronic supplementary material, appendix S1).

##### 4.1.1.2. Pliosauridae

The lower jaws of pliosaurids are the most diverse among all marine reptile groups, with coexistence of sympatric taxa. For example, there are two separated clusters of Callovian pliosaurids: the longirostrine taxa *Peloneustes* and *Marmornectes* on the one side, and large-bodied thalassophoneans (*Liopleurodon*, *Simolestes* and ‘*Pliosaurus*’ *andrewsi*) on the other (figures 2 and 3). This separation has both phylogenetic and ecological significance. Specifically, the two clusters are indicative of the different morphologies within the group [26,31,49,51,66,70–73] and closely mimic the split of pliosaurids between the Pierce and the Generalist guilds [5,9]. The main morphological differences between these groups are related to the robustness of the lower jaws and length of the mandibular symphysis (figures 5 , 7 and 8; electronic supplementary material, appendices S4 and S5). Longer and less-robust lower jaws, combined with relatively smaller adductor muscle masses, make *Peloneustes* and *Marmornectes* better suited than large pliosaurids to feeding on agile prey items [26]. These morphological features and biting styles provide functional support to their classification in the Pierce Guild. Conversely, large-bodied thalassophoneans (*Simolestes*, *Liopleurodon*, ‘*Pliosaurus*’ *andrewsi* and *Pliosaurus* spp.), have comparatively larger muscle adductors, and more effective bite transmission. Perhaps surprisingly for animals their size (table 3), they also have comparatively fast jaw-opening speed (high oMA values) (figure 6, electronic supplementary material, appendix S4). Thanks to their large size, however, taxa of the Generalist guilds are better suited to deal with hard prey (i.e. other marine reptiles) than their smaller-bodied relatives in the Pierce Guild. We confirm a previously noticed wide variety of functional morphologies within the genus *Pliosaurus* [26], as revealed by the broad PCoA space occupation of this genus (figures 3 and 5). The selective disappearance of small-bodied pliosaurids in the JSBS (supported by statistically significant values in the *t*-tests, for ML), across the Oxfordian–Kimmeridgian boundary (figure 3), results in the fact that only pliosaurids present in the KCF are overall bigger, and only macropredators belonging to the Generalist guilds.

### 4.1.2. Thalattosuchia

#### 4.1.2.1. Metriorhynchidae

Metriorhynchidae in Metriorhynchidae, metriorhynchines and geosaurines occupy close but scarcely overlapping areas of the functional morphospace. Their separation mainly occurs along PCo2 and PCo3, with metriorhynchines occupying smaller values along those axes, primarily due to their long gracile lower jaws and comparatively shorter maL/ML (figure 2). Interestingly, metriorhynchines have an equally faster jaw-opening profile but, unsurprisingly worse transmission at the biting position compared with geosaurines, which have deeper jaws better suited to resisting higher stresses during feeding (figures 6–8). Geosaurines jaws are, in fact, generally larger, more robust (i.e. with shorter and deeper symphyses) and have larger adductor musculature and higher TI values than metriorhynchines (figures 5, 7 and 8). This pattern peaks in Late Jurassic geosaurines of the Cut Guild. *Geosaurus*, *Plesiosuchus*, and *Dakosaurus* (GPD-clade) have the most effective bites among the marine reptiles in the JSBS (highest MA values behind macrophagous teleosauroids, and comparable to that of large-bodied pliosaurids), and jaw shape that is well suited to resist the stresses associated with macrophagous feeding (Cut Guild) (table 3; figures 5–8; electronic supplementary material, appendix S5). Interestingly, the same features are also present in all their Middle Jurassic sister taxa, even before these groups acquired their distinctive tooth adaptations. Indeed, the lower jaws of Middle Jurassic geosaurines of the GPD-clade (figure 3), such as NHMUK PV R 3321 (currently being described), *Suchodus* and *Ieldraan* do not significantly differ functionally or morphologically, except perhaps in their smaller size, from their Late Jurassic closest relatives *Dakosaurus*, *Plesiosuchus* and *Geosaurus*, respectively (figures 6 and 7 electronic supplementary material, appendix S5). This means that, unlike in pliosaurids, the Middle–Late Jurassic transition does not coincide with a drastic functional shift in lower jaws mechanics in metriorhynchids (electronic supplementary material, appendices S4 and S6), as demonstrated by the fact that these groups maintained their occupied area in the PCoA (figure 3). This result contrasts with qualitative observation [17] and quantitative testing of the dentition of these taxa, which determines a clear shift in Guild occupation, with OCF species in the Pierce Guild, and KCF taxa predominantly occupying the Cut Guild [5]. This result further highlights the importance of serration morphologies in marine reptile ecology [5,17,51]. This result is perhaps unsurprising, as macrophagous adaptations in the lower jaws and teeth are diagnostic of the whole clade [74].

The limited sample size makes it impossible to determine statistically significant differences between taxa of the T*-*clade and GPD-clade; however, the lower jaw functional morphologies of these groups appear to differ in some key aspects (figures 7 and 8; table 3). Species closely related to *Torvoneustes* in the Crunch Guild, have comparatively smaller jaw adductor musculature (maL/ML) and teeth (TI1), alongside slower and less-efficient bites (lower MA and higher oMA values), than their relatives GPD-clade in the Cut Guild (figures 7 and 8; table 3).

Overall, there is an increase in biting performance between metriorhynchines and geosaurines, with taxa of the GPD-clade having high MA values—only second to machimosaurins and pliosaurids. The increased biting performance in Late Jurassic metriorhynchids is associated with (i) an increase in size; (ii) deepening and shortening of the mandibular symphysis; (iii) enlarged muscle insertion sites compared with the basal most geosaurine taxa *‘Metriorhynchus’ brachyrhynchus* and *Tyrannoneustes lythrodectikos* (figures 7 and 8; table 3). It has been qualitatively and quantitatively observed that derived geosaurines are characterized by mandibular morphologies that increased their optimum gape angle, which would have reduced their bite force [29,74]. However, previous studies have also shown that geosaurines evolved: (i) shearing occlusion mechanics and (ii) contiguous, well-developed serrations [17,51]. These dental/occlusion specializations might have reduced the need for higher bite forces, and we demonstrate that dental features lagged behind (or even evolved without) specific mandibular specialization (e.g. in *Torvoneustes* clade), as it occurred in Machimosaurini.

#### 4.1.2.2. Teleosauroidea

Teleosauroids are also split into two distinct areas of the morphospace, indicating very different functional mandibular morphologies (figures 2 and 6–8). The longirostrine teleosauroids (i.e. the machimosaurid *Charitomenosuchus leedsi* and the teleosaurid *Mycterosuchus nasutus*) have jaw mechanics similar to other members of the Pierce II Guild (e.g. metriorhynchines); on the other extreme, Machimosaurini and their closest relatives (*Neosteneosaurus edwardsi* and *Proexochokefalos heberti*) are widely distinct from all other groups along PCo2 because of their enlarged muscle insertion sites (comparatively the most developed in all marine reptiles) (figure 3). This latter feature is so extreme that machimosaurins have the most effective bite (i.e. highest values for ltMA) (figures 5–8). This makes mechanical sense because an increase in the in-lever arm length (~maL), increases the value of the mechanical advantage ratio. The increase in maL is so dramatic that it counterbalances the considerable ML of these taxa (all teleosauroids have comparatively long mandibular symphysis, which by increasing the out-lever arm tends to reduce MA, although patterns of mandibular shortening are seen in the clade [27]. However, the trade-off for high bite efficiency is a lower jaw-opening velocity compared with longirostrine taxa, such as *Mycterosuchus nasutus* and *Charitomenosuchus leedsi* (figures 7 and 8; table 3; electronic supplementary material, appendix S4). The sharp dorsal curvature of the posterior half of machimosaurin lower jaws (absent in longirostrine teleosaurids and convergent with derived geosaurines), is probably an adaptation to (i) increase the size of the muscle insertion sites; (ii) re-orientation of the M. pterygoideus muscle group line of action; and (iii) increase gape. Although the teeth of both *Neosteneosaurus edwardsi* and *Proexochokefalos heberti* indicate they belong to the Pierce Guild together with their gracile longirostrine relatives, their jaws are functionally more similar to Machimosaurini of the Crunch Guild (figures 5–8; electronic supplementary material, appendix S5)—a pattern that was also found by Johnson *et al*. [27]. As highlighted above, this result indicates a decoupling in tooth morphology and lower jaw evolution in macrophagous teleosauroids [75], with the former lagging behind the latter, as it is also observed in macrophagous geosaurines and pliosaurids [27,70]. Across the Middle–Late Triassic boundary, there is a loss in taxonomic diversity of teleosauroids, but both functional extremes are still preserved, and exemplified by the diversification of *Machimosaurus* and the persistence of longirostrine forms such as *Bathysuchus megarhinus* (a taxon that is unfortunately too incomplete to be included in this dataset) (76 [5].

### 4.1.3. Ichthyosauria

Ichthyosaurians of the JSBS are functionally similar to other members of the Pierce I Guild (i.e. metriorhynchines and longirostrine teleosauorids). This similarity is demonstrated by the proximity of these taxa in the PCoA, and through the comparison of their morphology and bite mechanics (figures 2, 3, 5 and 6; table 3; electronic supplementary material, appendix S5). In fact, the very features that characterize Pierce Guild members (low ltMA, small maL/ML) are seen reaching extremes in ichthyosaurians. These animals have comparatively the least efficient bite of all marine reptiles in our dataset combined with the fastest velocity of jaw opening (figures 6–8). Ichthyosaurian jaws combine the smallest relative adductor muscles attachment sites, with relatively gracile and long jaws (figures 4, 6 and 7). Overall, their slender jaws are mechanically the best suited to catch soft and agile prey (e.g. squids and fast-moving fish), consistent with their tooth morphology (e.g. low TI) and implantation (aulacodont tooth attachment = teeth set in groove) [5,9].

The Middle–Late Jurassic transition witnessed the appearance of two (perhaps three) new types of lower jaw shapes in the Smash Guild (figure 5; electronic supplementary material, appendix S4). The first is that of *Bachypterygius extremus* which is larger and more robust than *Ophthalmosaurus* [31,77] but essentially maintains the same jaw functional mechanics. The lower jaw of *Bachypterygius extremus* is, interestingly, functionally similar to the metriorhynchines members of the Pierce Guild along PCo1 (figure 2). It is plausible that the different tooth morphology (size and wear), or more plausibly, habitat partitioning (i.e. ichthyosaurians have deep-diving adaptations absent in other groups) [17], is indicative of distinct diet preferences between these taxa [78]. The second type is represented by two undescribed Tithonian taxon from the MJML collection. Both specimens (MJML K1747 and MJML K1009) are considerably smaller than *Ophthalmosaurus*, and have even lower bite efficiency than both of their close relatives in our dataset. However, what these taxa lose in bite force, they gain in jaw-opening speed, having the lowest oMA values of the dataset (figures 6–8; table 3; electronic supplementary material, appendix S5); *Thalassodraco etchesi* [79] is a noticeable example of this group. It is unclear where *Nannopterygius enthekiodon* would fall, because this taxon is also too incomplete to confidently measure most of the functional metrics.

### 4.2. Functional morphology, tooth guilds and prey preferences

The statistically significant separations among taxonomic groups in the PCoA (figures 2 and 3; electronic supplementary material, appendix S3) are consistent with the hypothesis that marine reptiles partitioned food resources based on morphofunctional complexes and thus minimized competition for available prey. Overall, with minor discrepancies, the results of this work are broadly consistent with qualitative anatomical observations, diet inferences and ecological classification based on tooth morphology [5,9].

Specifically, all macrophagous marine reptiles (Late Jurassic geosaurines and pliosaurids) are characterized by enlarged adductor musculature, shortened mandibular symphyses (in absolute size, but also relative to their immediate outgroups), robust jaws and medium to large dimensions [17,19,32,51,78] (figures 6 and 7). These features contribute to efficient bites, but generally at the cost of a reduced jaw-opening speed [21]. Within macrophagous groups, the main functional difference between taxa of the Cut Guild (Geosaurines) and Generalist guilds (large-bodied pliosaurids) is the larger size of the latter (figure 6). Interestingly, though large-bodied pliosaurids also have comparable bite speed to that of the much smaller geosaurines (figure 6), confirming that, in order to fill in apex predator niches, size is a more important factor than other anatomical adaptations (i.e. tooth shape) [4]. This result is consistent with the comparative biomechanical testing of the mandibles of large-bodied pliosaurids *Pliosaurus kevani* and *Kronosaurus queenslandicus* compared with that of modern crocodylians [32,38,52]. At the same time, specific morphological features in geosaurines contribute to increase their functional performances, without the need for achieving gigantic size. For instance, compared with their immediate outgroup, geosaurines have more robust, and posteriorly curved mandibular rami. This specific feature, also present in Machimosaurini, modifies the line of action of adductor musculature, allows for an increase in its size, and is often seen in concert with wider optimum gape angles, which, in turn, reduces absolute bite force [51,74].

Foffa *et al.* [5] hypothesized that tooth morphology alone cannot be used to distinguish further subdivisions within the Pierce Guild (= Pierce I, mainly occupied by plesiosauroids; and Pierce II including longirostrine teleosauroids, metriorhynchids and small-bodied longirostrine pliosaurids), as originally proposed by Massare [9]. Instead, this study shows how taxa of the Pierce I and Pierce II Guilds of the OCF and KCF are instead characterized by drastically different lower jaw functional morphologies. On one side, plesiosauroids of the Pierce I sub-Guild share slow jaw opening combined with poorly effective bites and the highest TI of our sample (figures 5–8). Furthermore, these taxa do not have anteriorly developed mandibular symphyses, and have the smallest jaws compared with the body size of all marine reptiles. This combination of features indicates that plesiosauroids probably adopted a different feeding strategy (i.e. filter/sieve/straining feeding, raking sediments [15,33,67] or ‘Trap Guild’ [68] than other members of the Pierce Guild. In Pierce II sub-Guild, small-bodied pliosaurids, longirostrine teleosauroids and metriorhynchines are characterized by relatively weak force transmission to the bite point, have gracile jaws, with long protruding mandibular symphyses and, crucially, fast-opening jaw mechanisms (figures 5–8; electronic supplementary material, appendix S5). This combination of characters made these animals well suited to catch fast-moving prey, such as small fish and cephalopods. Modern analogues adopting this feeding strategy are river dolphins and gharials, while plesiosauroids could be better compared with some marine turtles or potentially to bottom-feeder sirenians (although bottom feeding in sirenians is often achieved through the aid of elaborate soft tissue features that cannot be assessed in plesiosauroids).

Ichthyosaurians in the Smash Guild adopted an analogous feeding strategy to the Pierce II sub-Guild. The shared features among metriorhynchines, longirostrine teleosaurids and ichthyosaurians, indicate that the transition between guilds can sometimes be difficult to determine, and there is a mechanical continuum among groups. However, the very features that characterize the Pierce (II) Guild are brought to an extreme in ichthyosaurians. Compared with the taxa belonging to the Pierce Guild, ichthyosaurians have considerably lower oMA values (faster opening mechanisms), longer mandibular symphyses and comparatively lower TI (figures 5–8; table 1). Overall, consistent with gut contents, body plan and tooth morphology, this combination of features support that ichthyosaur jaws were also well suited to catch fast-moving prey, such as fish and cephalopods.

Within Teleosauroidea, Machimosaurini were a group of large-bodied, derived teleosauroids, with blunt, serrated and heavily ornamented teeth, and have been linked to a durophagous diet [13,27,70,80]. Our results show that these taxa (*Lemmysuchus obtusidens* and *Machimosaurus*) were indeed functionally distinct from their longirostrine relatives (e.g. *Charitomenosuchus leedsi* and *Mycterosuchus nasutus*) in the Pierce Guild. *Machimosaurus* and *Lemmysuchus* jaws are, in fact, considerably more robust, and their adductor muscles are larger [27] than those of their relatives. The posterior halves of machimosaurin mandibles are strongly curved in the dorsal direction, increasing the insertion site for the M. pterygoideus muscle group and modifying their lines of action. This set of features is shared—although not as extreme—with some geosaurines of the T-clade (i.e. *Torvoneustes* sp. and Mr. Passmore’s specimen OUMNH J.1583, cf. *Metriorhynchus hastifer*). Based on shared characteristics of their dentition, machimosaurins, Torvoneustes and OUMNH J.1583 are assigned to the Crunch Guild. However, they have different morphofunctional features (figures 5–8) and thus cluster in distinct areas from machimosaurins in our PCo plot (figures 2 and 3). It is possible that the taxa in the T-clade could feed on hard-shelled preys without substantially modifying the ancestral feeding mechanics inherited from basal geosaurines.

### 4.3. Resource partitioning in extant and extinct multi-clade communities

Our understanding of the diversity of marine tetrapods is strongly biased by Lagerstätte effect, due to the intensive collecting of a limited number of high-diversity assemblages [81]. Numerous multi-clade assemblages exist through the Mesozoic and beyond: among many others, the Early Triassic Chaohu Fauna, and the Middle Triassic faunas of Xingyi and Monte San Giorgio are home of diverse marine reptiles ranging from placodonts, eosauropterygians, thalattosaurs, ichthyosaurs and sauropterygians [82,83], including sympatric closely related species [84] the Jurassic Blue Lias Formation and Posidonia Shale [85] include a variety of ichthyosaurs, thalattosuchians and plesiosaurs; the formations of Cretaceous Western Interior Seaway yield diverse assemblages of plesiosaurs, mosasauroids and turtles. This abundance of taxonomically diverse assemblages, extinction and turnover of lineages [9,11,13,16,18,23,24,81,86–94] and appearance of morphological innovations throughout the Mesozoic underlines the frequency of multi-clade communities in the fossil record. Similarly, the range of size, craniodental shape and body plans of the members of these faunas hints that the coexistence of such rich assemblages may have been facilitated by partitioning of habitat and resources [9,87,88,90,95], as hypothesized in modern ecosystems.

However, the lack of direct data, the scarcity of sufficiently well-preserved assemblages and rarity of approaches that are applicable to a disparate selection of lineages make the idea of partitioning resources difficult to test in extinct multi-clade communities. Despite the abundance of potential case studies [81], the idea of niche partitioning has been commonly invoked to explain the co-occurrence of multiple tetrapod lineages in rich aquatic faunas, primarily based on observation of taxonomic diversity rather than quantitatively tested at assemblage level. The rarity of such comparative approach hinders our understanding of the short- and long-term macroevolution, patterns of ecological turnover and diversity in aquatic ecosystems.

Famously, Massare [9] discussed the partitioning of diverse Jurassic and Cretaceous marine reptile assemblages based on swimming capability and the comparison of extinct tetrapod teeth with those of modern ones, for which dietary data are known. Ever since attempts to quantitative testing morphofunctional features that could indicate the partitioning of resources have primarily focused on broad-scale macroevolutionary studies regardless of the effective coexistence of taxa [10]. These include numerous studies on different lineages of ichthyosaurs, plesiosaurs, thalattosuchians and mosasaurs [26,27,78,96,97]. Only rarely the sympatry of taxa has been considered. For instance, stable isotope and dental microwear studies have been famously used to reveal differing foraging habitat and prey preferences in type-Maastrichtian mosasaurs [98–100], and similarly, habitat segregation has been suggested for mosasaurs of the Upper Cretaceous of Alabama [101]. Differing dietary preferences in mosasauroids are mimicked by craniodental morphofunctional differences [96,97]. In light of these studies, while we have a growing grasp of the macroevolution of marine tetrapods at lineage levels, our understanding of the evolution and structure of aquatic tetrapod communities is more fragmentary.

The partitioning of resources in modern marine multi-clade communities/assemblages is better understood and is supported by direct observations on foraging habits, diving capability and habitat preferences [102–107], geochemical data (e.g. 108) and dental microwear analyses [109]. Crucially, Kelley and Motani [6] unlocked a fundamental step to quantitatively test the idea of resource partitioning in the fossil record, by quantitatively linking craniodental morphological data with dietary preferences in modern aquatic tetrapods. Further steps in this direction have been taken by quantitatively comparing craniodental features of extinct clades alongside those of modern tetrapods [4,10,11,96,97].

In this context, this study provides a unified approach to tackle the question of niche partitioning in multi-clade aquatic ecosystems. In doing so, we add quantitative support to the idea that extinct aquatic tetrapod ecosystems were also characterized by a structured partitioning of food resources by means of morphofunctional differentiation among coexisting lineages. Specifically, we suggest that the segregation of morphofunctional craniodental characteristics facilitated—alongside habitat preferences, diving habits, among others—the coexistence of diverse multi-clade aquatic tetrapod assemblage throughout and beyond the Mesozoic. The ever-present diversity of multi-clade aquatic tetrapod communities since the early Triassic hints that this may be the case, with a growing body of qualitative and quantitative data to back this hypothesis. However, more in-depth studies that focus on marine ecosystems at the community/assemblage level are needed to test this hypothesis, ideally combining morphofunctional, geochemical and microwear analyses.

## 5. Conclusion

This study investigates the functional morphology of sympatric marine reptiles using a quantitative and comparative approach. Marine reptiles of the JSBS are morphologically and functionally distinct, as different phylogenetic groups cluster separately from the others with minimal overlap. Nonetheless, similar combinations of morphological characters and analogous biting performances are shared by unrelated taxa in the same tooth-based feeding guilds. Our analyses of lower jaw functional morphology are unable to differentiate between some guild groups. These results indicate that tooth morphology (specifically presence/absence of serrations) is a key feature for these ecological classes. In contrast, our analysis distinguished two distinct groups of jaw shapes associated with the Pierce Guild (Pierce I and Pierce II). The peculiar biomechanics of plesiosauroid jaws (small size, lower force transmission and low opening velocity) support the idea that these animals were better suited to low-stress foraging (filter, sieve or racking feeding), compared with metriorhynchines and ichthyosaurs in the Pierce and Smash guilds. We highlight at least two instances of mandibular combinations pre-dating the development of tooth shape characteristics of the Crunch, and Cut guilds: in the teleosauroids, where *Neosteneosaurus edwardsi* and *Proexochokefalos heberti* share similar mandibular features of durophagous machimosaurins without sharing the same characteristic crunching tooth morphology; and in metriorhynchids, where the jaws of basal geosaurines remain largely unmodified in most derived clades.

Our results of the PCoA broadly mimic the patterns previously found in our previous study of tooth disparity. Shared results are: (i) the faunas of OCF and KCF are markedly distinct taxonomically and morphofunctionally; (ii) small-bodied pliosaurids and Late Jurassic thalassophoneans are morphofunctionally distinct; (iii) the two main metriorhynchid groups (metriorhynchines and geosaurines) are morphofunctionally distinct; within geosaurines members of the T-clade broadly maintain the ancestral condition, while taxa in the GPD-clade evolve towards more robust and bite-efficient jaw shapes; (iv) longirostrine teleosauroids and machimosaurins are morphofunctionally distinct, with the already mentioned notable exception of *Proexochokefalos heberti* and *Neosteneosaurus edwardsi*; and (v) ichthyosaurians and plesiosauroids maintain their morphospace occupation through time.

However, we reveal that distantly related groups converge to similar feeding strategies through broadly similar morphofunctional patterns. For instance, non-machimosaurin teleosauroids, small-bodied pliosaurids, metriorhynchines and ichthyosaurians—all inferred to feed on small agile prey items—have shallow, fast-opening lower jaws, with long mandibular symphyses and relatively lower force transmission at the bite position. In contrast, macrophagous taxa are characterized by increased absolute bite force and higher mechanical resistance. These features are achieved either through morphological adaptations, or sometimes by sheer increase in body size. In the Crunch Guild, the convergence between machimosaurin teleosauroids and some geosaurines is limited to tooth morphology, and the two clades are functionally distinct. In light of these discrepancies, we demonstrate that lower jaw morphofunctional characters are better predictors of taxonomic groups than of tooth-based feeding guilds.

Overall, this study indicates partitioning of functionally important morphological features, which along with dentition specialization may have been a driver of niche partitioning and could have been an important factor facilitating the existence of highly diverse marine reptile assemblages over time. Nevertheless, more studies that specifically focus on marine ecosystems at the community/assemblage level are needed.

## Data Availability

The datasets supporting this article have been uploaded as part of the electronic supplementary material [110].
